# Feasibility and Preliminary Efficacy of an Online Cardiovascular Disease Prevention Randomised Controlled Trial Targeting Addictive and Compulsive Overeating Among Australian Young Adults

**DOI:** 10.1111/jhn.70102

**Published:** 2025-07-28

**Authors:** Mark A. Leary, Janelle A. Skinner, Melinda J. Hutchesson, Megan Teychenne, Megan C. Whatnall, Phillipa J. Hay, Tracy L. Burrows

**Affiliations:** ^1^ School of Health Sciences, College of Health, Medicine and Wellbeing University of Newcastle Callaghan New South Wales Australia; ^2^ Food and Nutrition Program, Hunter Medical Research Institute University of Newcastle New Lambton Heights New South Wales Australia; ^3^ School of Exercise and Nutrition Sciences, Institute for Physical Activity and Nutrition (IPAN) Deakin University Geelong Victoria Australia; ^4^ Translational Health Research Institute Western Sydney University Campbelltown New South Wales Australia; ^5^ Mental Health Services Campbelltown New South Wales Australia

**Keywords:** addictive eating, cardiovascular disease, young adults

## Abstract

**Background:**

Cardiovascular disease (CVD) is a major cause of mortality and morbidity. The number of young adults with at least one CVD risk factor has increased over the past two decades. Evidence suggests that addictive eating behaviours cluster with other CVD risk behaviours, including poor diet, lack of physical activity and poor sleep quality. The primary aim of this pilot study is to determine the feasibility (adherence to the programme), acceptability and engagement of an innovative intervention comprising of five telehealth sessions ranging from 20 to 45 min with an Accredited Practising Dietitian over 8 weeks aimed at improving CVD risk factors in young Australian adults aged 18–35 years with addictive eating behaviours.

**Methods:**

A total of 53 young adults with ≥ 3 symptoms of addictive eating assessed via the Yale Food Addiction Scale 2.0 and a body mass index > 18.5 kg/m^2^ were recruited. Adherence to the programme was assessed through the number of participants completing the telehealth sessions. Acceptability (including overall satisfaction, appropriateness and effectiveness of recruitment materials) was assessed via semi‐structured phone interviews with participants upon study completion. Engagement with the programme website was assessed with Wix Analytics and comprised of website access and webpage dwell time. Changes in dietary intake, food addiction symptoms, physical activity, sleep quality, mental health (depression, anxiety and stress), and CVD blood biomarkers were also assessed and compared between groups and over time.

**Results:**

Nine of the 27 participants randomised to the intervention group did not adhere to the programme by completing all five telehealth sessions. Twenty‐one (56.8%) participants completed the semi‐structured phone interview, with all reporting an overall ‘positive’ experience with the programme. Based on the total number of participants provided with website access in the intervention (*n* = 21) and control (*n* = 16) groups, the percentage who logged in to the website at least once was 95.2% and 18.8% respectively. The intervention group was superior compared to the control group for improvements in percent from fat (−4.87%/day [95% CI: −8.04, −1.70], *p* = 0.005), saturated fat (−1.77%/day [95% CI: −3.33, −0.20], *p* = 0.03) and non‐core foods (−9.20%/day [95% CI: −17.53, −0.87], *p* = 0.03) from baseline to 8 weeks. While other outcomes (i.e., changes in food addiction symptoms, physical activity, sleep, mental health, stress and CVD biomarkers – triglycerides) improved significantly over time, there were no between‐group differences.

**Conclusions:**

A CVD intervention targeting addictive eating behaviours successfully recruited young adults, a recognised population group difficult to reach. The telehealth programme was found to be feasible and demonstrated improvements in dietary outcomes.

## Summary

- Addictive eating behaviours often cluster with other CVD risk behaviours, including poor diet, lack of physical activity and poor sleep quality.
- The online ‘TRACE programme – young adults’, which aimed to improve CVD risk factors in young adults, was found to be feasible.
- A larger, more appropriately powered RCT is now needed to examine the efficacy of the TRACE programme at improving CVD risk factors in young adults.

## 1 Introduction

Cardiovascular disease (CVD) is a major cause of mortality and morbidity [1]. In Australia, an estimated 4.5 million people are living with CVD, resulting in approximately 120 deaths per day or 43,800 deaths annually [2]. The number of young adults with at least one CVD risk factor (namely increased weight, diabetes incidence and engaging in CVD risk behaviours such as poor nutrition and reduced physical activity) has increased [1]. Many young adults, often defined as 18–35 years, are unaware that they have risk factors and may exhibit lower health literacy [3]. Therefore, increasing the knowledge and individual awareness of CVD risk factors in young adults is beneficial to help reduce the risk of developing CVD.

There are many reasons why young adults experience increased CVD risk factors. It is a key life stage where many young adults move out of home, start university and enter the workforce [4]. These transitions can lead to changes in their health behaviours. Previous studies demonstrate that young adults engage in unhealthy behaviours which often cluster together, increasing health risks (such as smoking, excess alcohol consumption and poor nutrition) compared to other population groups [5, 6]. Young adults report sub‐optimal nutrition and physical activity behaviours [7], yet often do not seek help, which may contribute to weight gain. Therefore, it is important to promote healthy habits during this time to prevent chronic diseases such as obesity and CVD [8, 9]. Current nutrition guidelines for CVD focus on decreases in saturated/trans‐fat, as well as encouraging healthy eating patterns such as the consumption of vegetables, fruit, wholegrains, and healthy fats [8]. These nutrition guidelines, however, do not address eating behaviours such as regular meals or why people eat (e.g., mood‐related eating, including emotional and stress, or using food to cope with mental health conditions such as depression and anxiety) [10]. Despite the extent of obesity and CVD, research arising from interventions to manage CVD risk factors is only moderately effective in the short term [11]. This may be due to a range of reasons including lifestyle interventions (involving exercise and dietary elements) being met by participants with a range of barriers, such as lack of time to exercise and prepare healthy meals [12], lack of personalisation [13], as well as lack of support and continuity thus reducing the long‐term efficacy of the intervention [14].

Addictive eating is commonly characterised by the excessive and uncontrolled consumption of certain foods, particularly energy‐dense hyper‐palatable foods or ultra‐processed foods [15, 16, 17], and is operationalised using the Yale Food Addiction Scale (YFAS) [18]. Symptoms of addictive eating include craving, loss of control, tolerance and withdrawal associated with eating behaviours, with approximately one in six young adults experiencing addictive eating [19]. Although not currently recognised as an official disorder in the Diagnostic and Statistical Manual of Mental Disorders (DSM‐5) [20], addictive eating is associated with mental ill‐health such as depression, anxiety and disordered eating [21], and has been found to contribute to poor dietary intake [22, 23, 24], poor sleep quality [25], increased weight status [26], and to be a key risk factor for CVD [27].

Addictive eating behaviours have been recognised as complex and multifaceted that requiring a comprehensive approach for effective intervention [25, 28, 29]. Interventions that target multiple health behaviours are needed to address the underlying causes and help young adults develop healthy coping mechanisms and lifestyle habits, which may provide a protective effect against CVD or improve CVD risk outcomes. To the authors' knowledge, there are no interventions targeting addictive eating in young adults [30, 31, 32, 33, 34, 35, 36, 37, 38, 39, 40, 41]. This study builds on evidence including the co‐design of the TRACE (Targeted Research on Addictive and Compulsive Eating) programme [42], and an efficacious randomised controlled trial in adults ≥ 18 years, which reported improvements in addictive eating symptoms, mental health outcomes and diet quality for participants who engaged in the TRACE intervention [25, 28]. As such, the TRACE programme, which offers a harm reduction, personalised approach for the management of addictive eating through support and education, has been further co‐designed and refined/developed with young adults with lived experience and health professionals to better support self‐efficacy in young adults. Results of the co‐design with young adults identified a need to refine the participant workbook, which included reducing the word length, content restructuring, and making the workbook only available as an online PDF version. Further recommendations from the young adults included the use of text messages between telehealth sessions to remind participants in the intervention group to complete activities and the addition of a strength training question to the physical activity questionnaire.

The primary aim of the current study is to determine the feasibility (programme adherence), acceptability (including overall satisfaction, appropriateness and effectiveness of recruitment materials) and engagement of the ‘TRACE Programme – young adults’ a telehealth intervention targeting CVD risk factors (diet, food addiction [FA] symptoms, physical activity, sleep quality and cardiometabolic profiles) in young Australian adults aged 18–35 years with addictive eating behaviours.

## 2 Materials and Methods

### 2.1 Study Design

The ‘TRACE programme – young adults’ was an 8‐week, non‐blinded, pilot randomised controlled trial with two parallel arms. Participants were randomly allocated to either (1) intervention or (2) control groups. All participants received detailed study information before providing informed electronic consent to participate. The study was approved by The University of Newcastle Human Research Ethics Committee (H‐2022‐0386) and was registered as a clinical trial with the Australian New Zealand Clinical Trials Registry before commencing recruitment (ACTRN12623001079639). The findings are reported in accordance with Consolidated Standards of Reporting Trials (CONSORT) guidelines for randomised pilot and feasibility trials [43].

### 2.2 Study Sample

A sample size calculation was not performed, as this was a pilot study. A total sample size of 50 (i.e., 25/group) was set based on available time for recruitment, study funding, and to allow for greater diversity within the sample by exceeding the median sample size of 30.5 among previous pilot trials [44, 45, 46]. Eligibility included (1) live in New South Wales (NSW) Australia, (2) proficient in English, (3) regular access to the internet, (4) aged 18–35 years, (5) endorse ≥ 3 FA symptoms, as measured via the YFAS 2.0 [47], (6) a body mass index (BMI) of 18.5 kg/m^2^ or more, (7) not have medically diagnosed CVD, including hypertension, or dyslipidaemia, and (8) no history of a medically diagnosed eating disorder, for example, Binge Eating Disorder. Individuals who indicated pregnancy or lactation, or bingeing or purging on the Eating Disorder Examination Questionnaire Short Form (EDEQ‐S) [48] were excluded. Participants were recruited from the community via local and national advertising, including social media and aimed to have no racial or gender bias (e.g., Twitter, Facebook and Instagram advertisements) from September 2023 to April 2024.

### 2.3 Procedure

Once eligibility was established and consent provided, participants completed baseline surveys and a CVD biomarker assessment (see Section 2.6). These measures were also completed at 8 weeks follow‐up. Participants were then randomly allocated using a block design via a computer‐based programme (www.randomization.com; block design with block sizes of six) by a blinded researcher. Blinding of participants and dietitians to group allocation was not possible due to the nature of the intervention. Following randomisation, participants were informed of their group allocation via email and provided with a detailed description of what this entailed. At the end of the study, all participants from both groups were invited to participate in a 10–20‐min semi‐structured feasibility and acceptability phone interview with a researcher.

### 2.4 Intervention

#### 2.4.1 Intervention Group

The intervention is based on stages of change theory [49] (operationalised through the patient activation measure [50]) and utilises a harm reduction approach. The intervention uses personalised feedback, skill‐building exercises and goal setting to help individuals reduce CVD risk factors, including poor diet, insufficient physical activity, poor sleep quality and cardiometabolic biomarkers, through awareness raising of at‐risk eating behaviours (e.g., emotional eating, stress eating and addictive eating). The intervention is based on an individual's dominant trait/s associated with addictive behaviour risk (i.e., depression proneness, anxiety proneness, sensation proneness and/or impulsivity proneness), measured via the Substance Use Risk Profile Scale [51]. Depending on the dominant addictive behaviour trait, specific coping skill strategies are then incorporated into the goal‐setting process. The TRACE programme targets change using a multicomponent clinician‐led approach (telehealth sessions, programme workbook, programme website and scheduled text messaging). Consistent with brief intervention principles, baseline surveys were used to identify at‐risk behaviours and provide participants with personalised feedback. Intervention participants received five telehealth sessions that followed a prescribed clinician manual. The first two sessions were longer at approximately 45 min, with a gradual reduction in session time (e.g., session five being approximately 20 min). Sessions were facilitated by an Accredited Practising Dietitian with training in motivational interviewing (MI), patient activation and disordered eating. Participants were sent reminder text messages 24 h before each session, and text messages to complete assigned homework tasks following telehealth sessions two, three and four. Specific details regarding the content included in each of the telehealth sessions can be seen in Additional Table S1.

Where possible, telehealth sessions used open‐ended questions, MI, reflective listening, affirmations of participants' strengths and periodic summaries to help consolidate participants' thoughts and increase participant activation. Participants had access to a study‐specific website and a participant workbook. The website contained resources to compliment the telehealth sessions and relevant sections of a manualised participant workbook, encouraging reflection. The participant workbook consisted of five modules that mirrored the content of the five telehealth sessions, with co‐designed character stories to increase the relatedness of the materials. The workbook also contained between‐session activities.

A clinician manual was used to ensure standardised intervention delivery, and a record of session implementation using a standardised template was kept. Participant adherence to the intervention was assessed by a session attendance checklist completed by the dietitian. The dietitian administering the telehealth sessions monitored the completion of homework tasks and workbook activities at the start of telehealth sessions two to five. Assistance was also provided by the dietitian at this time if participants experienced any difficulties completing the homework tasks/activities. Additionally, to assist with adherence, on completion of each telehealth session, the dietitian emailed a personalised ‘Addictive Eating Action Plan’, completed on a standardised template, to the participant.

#### 2.4.2 Control Group

Participants received ‘usual care’ and were provided with links to online resources for healthy lifestyle practices (e.g., the Heart Foundation and Australian Guide to Healthy Eating) at study commencement (i.e., baseline). Participants then received a passive version of the intervention, which included the self‐guided workbook and access to the study website, after study completion (i.e., 8 weeks from baseline).

### 2.5 Primary Assessment and Outcomes

#### 2.5.1 Feasibility and Acceptability

Semi‐structured phone interviews (approx. 10–20 min) were conducted by an independent researcher to determine the feasibility and acceptability of the study protocol and intervention components (see Additional Table S2). Questions based on participants' overall satisfaction, and the appropriateness and effectiveness of recruitment and survey materials were asked of all (intervention and control group) participants who volunteered to participate in the phone interviews. Intervention group participants were asked additional questions related to the delivery and timing of telehealth sessions, programme content, competency of the facilitator, website, use of correspondence (text messaging/email), reason/s for not completing the programme (if warranted) and overall satisfaction with the intervention. Control group participants were invited to participate in the phone interviews before receiving the passive version of the intervention. Responses were recorded. Participants were remunerated with a gift voucher (value AUD20). Adherence to the programme and website usage was analysed for acceptability.

### 2.6 Secondary Assessment and Outcomes

Secondary outcomes included changes in CVD risk factors – dietary intake, FA symptoms, physical activity levels, sleep quality and CVD biomarkers. A description of the measures used to assess these CVD risk factors, and other health behaviours, is outlined below in Table 1, including the survey measures.

**Table 1:** Outcomes measure/instruments reported on within the current study and scoring.

Measure	Description
Demographics	17 demographic questions including age, self‐identified gender, self‐reported weight and height (converted to BMI), postcode, medical history and medication use. Postcode data were coded post data collection for socio‐economic position via the Socio‐Economic Indexes for Areas (SEIFA) scale [52]. Two further questions assessed tobacco, e‐cigarette and vape use.
CVD risk factors	
Dietary intake and quality	Assessed with the Australian Eating Survey (AES) – Heart [53]. This validated semi‐quantitative food frequency questionnaire (FFQ) assesses intakes of nutrients and foods related to CVD, such as those from the Mediterranean [54] and Portfolio diets [55]. This included food preparation methods and use of salt‐reduced foods. This tool has a reporting period of the previous 6 months; however was amended for shorter follow‐up in the current study. Foods are categorised into two groups according to the Australian Guide to Healthy Eating: (1) ‘core’ or ‘healthy nutrient‐rich foods’ (breads and cereals, fruits, vegetables, dairy products, lean meats/fish/poultry/eggs/nuts), and (2) ‘non‐core’ or ‘unhealthy nutrient‐poor foods’ (e.g., sugar‐sweetened drinks, packaged snacks, confectionery, baked sweet products, take‐away foods, fatty meats) [53]. Using the AES – Heart, the dietary outcomes measured included total energy (kJ), percent energy (%E) from fat, saturated fat, monounsaturated fat, polyunsaturated fat, non‐core foods, total trans fat, total sodium and fibre.
Addictive eating	Assessed with the Yale Food Addiction Scale (YFAS 2.0), a 35‐item self‐report tool providing a symptom score ranging from zero to eleven [47]. Severity of addictive eating was classified as mild (2–3 symptoms), moderate (3–4 symptoms) or severe (6+ symptoms).
Physical activity and strength training	Assessed with the validated Active Australia Survey (AAS) [56], which contains eight questions with an additional question added about strength training. This questionnaire asks about physical activities undertaken for recreation, active travel and household purposes in the past week. To avoid over‐reporting errors, daily durations of physical activity for a single activity type were truncated at 840 min (i.e., 14 h) and total weekly physical activity (across all types) was truncated at 1680 min (i.e., 28 h) as per guidelines [56]. To enable comparisons with National Physical Activity Guidelines of 150 min of moderate physical activity per week and ≥ 75 min of vigorous physical activity per week, inactivity was defined as (0 min of physical activity per week), insufficiently active (≥ 1 or ≤ 149 min of moderate physical activity per week or ≥ 1 or ≤ 75 min of vigorous physical activity per week), or sufficiently active (≥ 150 min of moderate physical activity per week or ≥ 75 min vigorous physical activity per week).
Sleep	The Pittsburgh Sleep Quality Index (PSQI) [57] was used to assess the global sleep quality score, which may range from zero to 21 (where > 5 indicates poor sleep quality).
Health behaviours	
Mental health	Depression, anxiety and stress symptomology were assessed with validated tools, including the Patient Health Questionnaire‐8 (PHQ‐8) [58], the Generalised Anxiety Disorder‐7 (GAD‐7) [59], and the Perceived Stress Scale – 4 (PSS‐4) [60], respectively, each tool was scored according to author guidelines with higher scores representing greater severity.
Disordered eating	Measured using the Eating Disorder Examination Questionnaire 6.0 (EDEQ‐S), a validated 12‐item questionnaire that assesses the presence of key eating disorder symptoms [45].
Trait/s associated with risk of addictive behaviour	The validated 23‐item Substance Use Risk Profile Scale (SURPS) [51] was used at baseline to determine participants' dominant personality trait/s (impulsivity proneness, sensation proneness, depression proneness and anxiety proneness).
Participant activation level	The Patient Activation Measure (PAM‐13) [61] was used to measure participants' confidence and readiness to change health behaviours. Scores range from 0 to 100, with higher scores indicating individuals have higher levels of activation.
Quality of life	Assessed via the validated self‐report five‐item tool, the EQ‐5D‐5L [62]. The EQ‐5D‐5L was analysed to produce an index score between 0 (state of death) and 1 (perfect health).
Healthcare utilisation	Assessed via the Consumer Services Receipt Inventory (CSRI) reflecting healthcare services used in the preceding 8 weeks [63].

#### 2.6.1 Misreporting of Energy Intake (EI)

Plausible reporters of EI were identified at baseline using the Goldberg cutoffs [64]. Misreporting was identified if the ratio of EI to basal metabolic rate (BMR) was above or below the calculated cut‐off. The Schofield Equation [64] was used to calculate BMR. Cut‐offs were determined using the method suggested by Black [64] and used a physical activity level estimate of 1.4 (sedentary/light PAL). Participants were classified as under‐reporters, over‐reporters or adequate reporters consistent with other studies [25]. No one was excluded from the analysis.

#### 2.6.2 CVD Biomarker Assessment

Following an overnight fast (i.e., no food consumed after 10 PM the evening before), participants had a venous blood sample collected by a trained phlebotomist at a NSW Health Pathology Collection Centre. Blood samples were analysed according to standard operating procedures for total cholesterol, high‐density lipoprotein (HDL), low‐density lipoprotein (LDL), triglycerides, HbA1c and glucose by the respective pathology laboratories. Participants were remunerated with a gift voucher (value AUD50) on completion of each blood test. Due to a delay in blood collection, some participants *n* = 8 (*n* = 5 intervention group, *n* = 3 control group) did not complete their blood tests before commencement.

### 2.7 Statistical Analysis

#### 2.7.1 Analysis of Feasibility and Acceptability

Adherence was determined based on the number of intervention participants who completed all five telehealth sessions within the scheduled timeframe (i.e., session 1 – Week 1, session 2 – Week 2, session 3 – Week 4, session 4 – Week 6 and session 5 – Week 8). Website usage was collated for the period of October 2023 to July 2024 and analysed via ‘Wix Analytics’ [65].

Basic qualitative content analysis [66] of the interview data was used to gain an understanding of the perspective of participants regarding feasibility and acceptability. This was undertaken by an independent researcher with no prior relationship with the participants. Answers to the questions (with the number of participants responding) were tabulated and described descriptively.

#### 2.7.2 Analysis of Changes in CVD Risk Factors and Health Behaviours

The analysis was intention‐to‐treat, including all randomised participants. All available data were used with no imputation of missing values at 8 weeks.

Following descriptive statistics, between‐group differences at baseline, for demographics, YFAS symptom scores, mental health and participant activation scores were assessed using *t*‐tests, chi‐squared, Fisher's exact test or analysis of variance, as appropriate.

Modelling: To determine the change in CVD risk factors and health behaviours, Linear Mixed Models were used with main effects for group (intervention and control) and time (treated as categorical at levels baseline, and 8 weeks), and the group‐by‐time interaction with an unstructured residual covariance structure to allow for correlation between the repeated measurements. The outcome effect is reported as the difference between means at baseline and 8 weeks, with a 95% confidence interval (CI) for the difference. BMI and mental health conditions were examined for possible moderating effects on the effect size of YFAS scores, and if significant, adjustment for them was carried out. Statistical significance was set at 0.05, and analysis was performed using STATA version 16.1 [67].

## 3 Results

### 3.1 Description of Study Participants

**Figure 1:**
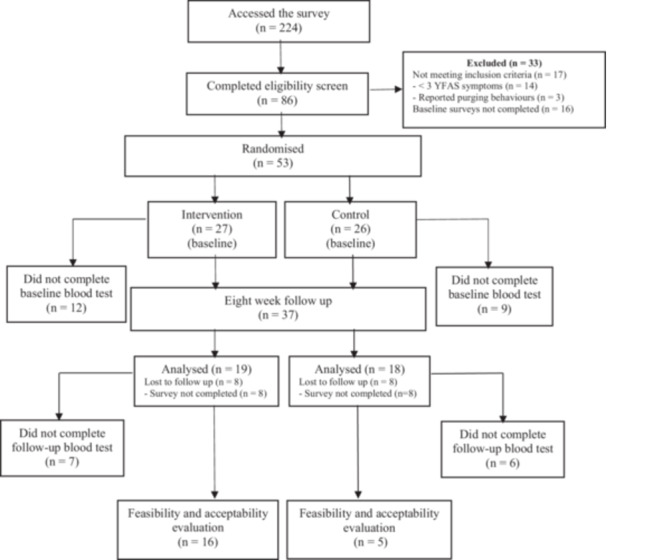
Flow diagram of participant recruitment and data analysis.

A total of 224 individuals accessed the survey, with *n* = 69 participants eligible and *n* = 53 randomised (*n* = 27 intervention, *n* = 26 control) (Figure 1). Of those randomised, the majority, *n* = 48 (90.6%), lived in a metropolitan area. There were no significant differences between groups at baseline (Table 2). The mean ± SD age of the total sample was 27.3 years ± 5.2; (range: 18–35 years), with 79.2% identifying as female (*n* = 42). The mean BMI was 29.2 kg/m^2^ ± 7.1 (range: 18.7–46.5 kg/m^2^). The majority of participants (*n* = 37, 69.8%) were classified as ‘severe’ for YFAS symptoms with a mean score of 7.2 ± 2.5 symptoms (range: 3–11). The mean global sleep score (PSQI) was 9.3 ± 3.1, indicating ‘poor’ sleep quality and the majority of participants (*n* = 45, 84.9%) reported activity that was classified as ‘sufficiently active for health’.

**Table 2:** Baseline characteristics (mean ± SD, number [*n*] and frequency [%]).

Variable	Intervention group (*n* = 27)	Control group (*n* = 26)	Total (*n* = 53)	*p*‐value
Age (years) (range: 18–35)	27.8 ± 4.9	26.8 ± 5.5	27.3 ± 5.2	0.50
Gender				0.11
Male^a^	8	3	11 (20.8%)	
Female	19	23	42 (79.2%)	
BMI (kg/m^2^) (range: 18.7–46.5)	28.7 ± 6.8	29.8 ± 8.4	29.2 ± 7.1	0.62
BMI category				
Healthy (18.5–24.99 kg/m^2^)	8	11	19 (35.8%)	
Overweight (25–29.99 kg/m^2^)	7	5	12 (22.6%)	
Obese (≥ 30 kg/m^2^)	12	10	22 (41.5%)	
Socio‐Economic Status (SEIFA) (range: 1–10)	6.3 ± 2.2	5.2 ± 2.6	5.8 ± 2.4	0.11
Patient activation measure (PAM‐13) category (range: 0–100)	58.7 ± 15.5	57.1 ± 13.0	57.9 ± 14.2	0.68
SURPS				
Anxiety proneness	13.1 ± 2.5	13.8 ± 2.6	13.5 ± 2.6	
Depression proneness	16.2 ± 4.1	16.7 ± 4.7	16.5 ± 4.3	
Sensation proneness	13.6 ± 3.3	13.6 ± 3.8	13.6 ± 3.5	
Impulsivity proneness	11.6 ± 2.6	12.2 ± 3.5	11.9 ± 3.0	
Depression (PHQ‐8) (range: 0–24)	9.6 ± 5.7	10.5 ± 5.5	10.0 ± 5.6	0.56
Anxiety (GAD‐7) (range: 0–21)	7.6 ± 4.8	9.1 ± 5.2	8.3 ± 5.0	0.30
Perceived stress (PSS‐4) (range: 0–16)	7.9 ± 2.3	8.7 ± 2.6	8.3 ± 2.4	0.23
Quality of life (EQ‐5D‐5L) (range: 0–100)	62.1 ± 18.1	65.1 ± 17.8	63.5 ± 17.8	0.55
Sleep Quality Global Score (PSQI) (range: 0 to 21)	9.7 ± 3.3	8.9 ± 2.9	9.3 ± 3.1	0.39
Physical activity total (h/week)^b^	7.55 ± 5.53	5.77 ± 4.78	6.68 ± 5.2	0.22
Inactive	2	1	3 (5.7%)	
Insufficiently active	1	4	5 (9.4%)	
Sufficiently active	24	21	45 (84.9%)	
Total FA symptoms (range: 3–11)	7.3 ± 2.7	7.1 ± 2.4	7.2 ± 2.5	0.76
Mild (2–3 symptoms)	3	3	6 (11.3%)	
Mod (4–5 symptoms)	6	4	10 (18.9%)	
Severe (≥ 6 symptoms)	18	19	37 (69.8%)	

CVD blood test	Intervention group (*n* = 15)	Control group (*n* = 17)	Total (*n* = 32)	Range
Total cholesterol (mmol/L)	4.9 ± 0.6	4.8 ± 1.0	4.8 ± 0.8	3.4–7.2
HDL‐C (mmol/L)	1.4 ± 0.3	1.4 ± 0.3	1.4 ± 0.3	0.8–2.0
Non‐HDL‐C (mmol/L)	3.5 ± 0.6	3.4 ± 0.9	3.5 ± 0.8	2.2–6.1
Total chol/HDL‐C ratio	3.8 ± 0.9	3.6 ± 1.0	3.7 ± 1.0	2.3–6.6
LDL‐C (mmol/L)	2.9 ± 0.5	3.0 ± 0.9	2.9 ± 0.8	2.0–5.7
Triglycerides (mmol/L)	1.4 ± 0.7	1.0 ± 0.4	1.1 ± 0.6	0.5–2.9
HbA1c – NGSP (%)	5.3 ± 0.5	5.0 ± 0.2	5.1 ± 0.4	4.6–6.4
Fasting glucose (mmol/L)	5.1 ± 0.3	4.7 ± 0.6	4.9 ± 0.5	3.6–5.7

Abbreviations: BMI, body mass index; FA, food addiction; HbA1c – NGSP, National Glycohemoglobin Standardisation Programme; HDL‐C, high‐density lipoprotein cholesterol; LDL‐C, low‐density lipoprotein cholesterol; SEIFA, Socio‐economic Indexes for Areas scale (1–10 with 1 being most disadvantaged and 10 being least disadvantaged).

^a^ *n* = 1 participant reporting ‘Other/non‐binary’ for gender randomly allocated to the male stratum.

^b^ Inactive (0 min per week), insufficiently active (≥ 1 or ≤ 149 min of moderate physical activity per week or ≥ 1 or ≤ 75 min of vigorous physical activity per week), sufficiently active (≥ 150 min of moderate physical activity per week or ≥ 75 min vigorous physical activity per week).

### 3.2 Primary Assessment and Outcomes

#### 3.2.1 Adherence to the Programme

Of the 27 participants randomised to the intervention, nine did not attend all five telehealth sessions, with six participants (22.2%) failing to start the programme, two participants (7.4%) completing the first two sessions only, and one participant (3.7%) completing their baseline blood test but failing to book their first telehealth session. Twenty participants (100%) from the intervention group that booked their first telehealth adhered to the programme schedules for sessions one and two (Week 1 and Week 2, respectively), followed by 15 participants (75%) for session three (Week 4), and 17 participants (85%) for sessions four and five (Week 6 and Week 8, respectively).

#### 3.2.2 Feasibility and Acceptability Interview

Twenty‐one participants (56.8%) completed the feasibility and acceptability interview (*n* = 16 intervention, *n* = 5 control). All participants reported their overall experience as ‘positive’, with two participants reporting difficulties organising blood tests. Sixteen participants from the intervention reported that the duration/availability of telehealth sessions was suitable, the information provided was appropriate and easy to understand, the website was easy to use, and the dietitian facilitating the sessions had good communication and was knowledgeable. The majority of participants (*n* = 9, 56.3%) reported that the number of telehealth sessions was sufficient, with 15 of the 16 participants (93.8%) also reporting that the workbook and text messages/email for correspondence were useful. Overall, participants rated their satisfaction with the intervention as ‘good’ or ‘very good’. Further details can be seen in Additional Table S1.

#### 3.2.3 Website Engagement

**Figure 2:**
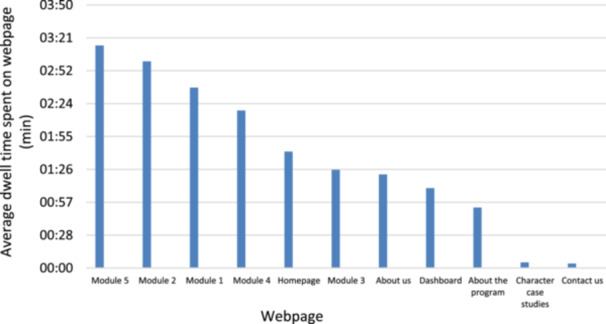
The average dwell time (minutes) spent on each webpage from October 2023 to July 2024.

Based on the total number of participants provided with website access in the intervention (*n* = 21) and control (*n* = 16) groups, the percentage who logged in to the website at least once was 95.2% and 18.8%, respectively, indicating a higher level of engagement by the intervention group. Across the time period (Figure 2), in descending order, the average dwell time per webpage was ‘module five’ (3:15 min), ‘module two’ (3:01 min), ‘module one’ (2:38 min), ‘module four’ (2:18 min), with the remaining webpages having a dwell time less than 2 min.

### 3.3 Baseline Dietary Intakes

The majority of participants (i.e., greater than 50%) did not meet National recommendations [68] except for %E from polyunsaturated fat and fibre intake for males (Table 3).

**Table 3:** Baseline diet characteristics (mean ± SD, number [*n*] and frequency [%]) compared to nutrient reference values for Australia and New Zealand [68].

Diet characteristics	Intervention group (*n* = 27)	Control group (*n* = 26)	Total (*n* = 53)	Australian nutrient reference values (NRVs)	Percent (%) meeting nutrient reference values (NRVs)
Total energy (kJ)	11,159 ± 5646	10,686 ± 4093	10,927 ± 4904	—	—
%E from fat	38.1 ± 5.6	40.9 ± 6.3	39.5 ± 6.2	20%–35% total energy intake	(*n* = 14, 26.4%)
%E from saturated fat	14.5 ± 2.4	15.6 ± 3.4	15.0 ± 3.0	< 10% total energy intake	(*n* = 2, 3.8%)
%E from monounsaturated fat	15.3 ± 3.0	16.8 ± 3.3	16.0 ± 3.2	< 15% total energy intake^a^	(*n* = 17, 32.1%)
%E from polyunsaturated fat	6.0 ± 1.9	6.3 ± 1.9	6.2 ± 1.9	< 10% total energy intake	(*n* = 51, 96.2%)
Total sodium (mg)	2891 ± 1682	2597 ± 990	2747 ± 1381	460–920 mg/day AI	(*n* = 1, 1.9%)
Total fibre (g)	28.1 ± 14.8	27.1 ± 11.9	27.6 ± 13.3		(*n* = 23, 43.4%)
Males	37.9 ± 22.3	40.8 ± 6.2	38.7 ± 18.9	30 g/day AI	(*n* = 7, 63.6%)
Females	24.0 ± 7.9	25.3 ± 11.4	24.7 ± 9.9	25 g/day AI	(*n* = 16, 38.1%)
%E from non‐core foods	41.1 ± 18.2	37.6 ± 12.2	39.4 ± 15.5	10%–15% (ADG)	(*n* = 2, 3.8%)

Abbreviations: ADG, Australian Dietary Guidelines; AI, adequate intake.

^a^ %E from monounsaturated fats is based on the subtraction of recommended intakes of saturated fats and polyunsaturated fats [69].

### 3.4 EI Misreporting

A sensitivity analysis of dietary intake was carried out and is provided in Additional Table S3. The majority of dietary changes remained statistically significant. Given the plausibility of answers and the high likelihood of decreased EIs post‐intervention, analysis with the entire sample was retained (Table 4).

**Table 4:** Changes in CVD risk factors and health behaviours within and between groups (baseline to 8 weeks with 95% confidence intervals).

	Difference over time			Difference between groups over time	
Variable	Intervention group	Control group	Time *p*	Intervention‐control	Group × time *p*
Nutrient					
Total energy (kJ)	−1460 (−2857, −64)	−957 (−2421, 507)	**< 0.05**	−503 (−2526, 1520)	0.89
%E from fat	−3.11 (−5.30, −0.93)	1.75 (−0.55, 4.05)	**< 0.05**	−4.87 (−8.04, −1.70)	**< 0.05**
%E from saturated fat	−1.61 (−2.69, −0.53)	0.15 (−0.98, 1.29)	**< 0.05**	−1.77 (−3.33, −0.20)	**< 0.05**
%E from monounsaturated fat	−1.48 (−2.52, −0.44)	0.72 (−0.38, 1.81)	**< 0.05**	−2.19 (−3.70, −0.68)	**< 0.05**
%E from polyunsaturated fat	−0.15 (−0.95, 0.65)	0.79 (−0.06, 1.63)	0.71	−0.94 (−2.10, 0.23)	0.31
Total trans fat (mg)	−367 (−698, −36)	−134 (−483, 216)	**< 0.05**	−233 (−714, 248)	0.65
Total sodium (mg)	−541 (−874, −208)	−200 (−549, 149)	**< 0.001**	−341 (−824, 141)	0.17
Fibre (g)	1.56 (−4.07, 7.18)	−1.69 (−7.57, 4.19)	0.59	3.25 (−4.89, 11.38)	0.44
%E from non‐core foods	−11.18 (−16.93, −5.44)	−1.98 (−8.01, 4.05)	**< 0.001**	−9.20 (−17.53, −0.87)	**< 0.05**
Food addiction					
Total FA Symptoms (range from 3 to 11)	−4.12 (−5.53, −2.70)	−3.60 (−5.05, −2.15)	**< 0.001**	−0.52 (−2.55, 1.51)	0.62
Sleep quality					
PSQI Global score (range from 0 to 21)	−2.02 (−3.41, −0.64)	−1.83 (−3.25, −0.40)	**< 0.05**	−0.20 (−2.18, 1.77)	0.84
Physical activity					
Physical activity total (min per week)	159 (53, 264)	97 (−11, 204)	**< 0.05**	63 (−87, 214)	0.42
Strength training (days per week)	0.47 (0.03, 0.91)	0.45 (0.01, 0.90)	**< 0.05**	0.02 (−0.61, 0.64)	0.99
Mental health					
Depression (range: 0–24)	−1.17 (−2.88, 0.54)	−1.15 (−2.91, 0.61)	0.18	−0.02 (−2.48, 2.43)	0.98
Anxiety (range: 0–21)	0.12 (−1.50, 1.74)	−1.21 (−2.87, 0.46)	0.88	1.33 (−0.99, 3.65)	0.54
Stress (range: 0–16)	−1.26 (−2.11, −0.41)	−1.45 (−2.32, −0.58)	**< 0.05**	0.19 (−1.03, 1.40)	0.96
CVD					
Total cholesterol (mmol/L)	−0.10 (−0.35, 0.15)	−0.17 (−0.42, 0.08)	0.43	0.07 (−0.28, 0.42)	0.70
HDL‐C (mmol/L)	0.05 (−0.11, 0.20)	−0.07 (−0.22, 0.08)	0.55	0.11 (−0.10, 0.33)	0.29
Non‐HDL‐C (mmol/L)	−0.13 (−0.36, 0.09)	−0.15 (−0.38, 0.07)	0.25	0.02 (−0.29, 0.34)	0.85
Total chol/HDL‐C ratio	−0.23 (−0.49, 0.04)	−0.10 (−0.36, 0.16)	0.09	−0.13 (−0.50, 0.24)	0.49
LDL‐C (mmol/L)	−0.05 (−0.29, 0.19)	−0.14 (−0.38, 0.10)	0.70	0.09 (−0.25, 0.43)	0.60
Triglycerides (mmol/L)	−0.23 (−0.44, −0.03)	−0.10 (−0.30, 0.09)	**< 0.05**	−0.13 (−0.41, 0.16)	0.40
HbA1c – NGSP (%)	−0.01 (−0.09, 0.08)	0.05 (−0.03, 0.14)	0.86	−0.06 (−0.18, 0.06)	0.33
Fasting glucose (mmol/L)	0.11 (−0.26, 0.48)	0.11 (−0.26, 0.48)	0.55	0.00 (−0.52, 0.53)	0.98

*Note:* Boldface indicates statistical significance.

Abbreviations: FA, food addiction; HbA1c – NGSP, National Glycohemoglobin Standardisation Programme; HDL‐C, high‐density lipoprotein cholesterol; LDL‐C, low‐density lipoprotein cholesterol; SEIFA, Socio‐economic Indexes for Areas scale (1–10 with 1 being most disadvantaged and 10 being least disadvantaged).

### 3.5 Secondary Assessment and Outcomes

Overall retention at 8 weeks was 69.8% (*n* = 37; *n* = 19 intervention group [70.4%], *n* = 18 control group [69.2%]). Changes in CVD risk factors and health behaviours are shown in Table 4.

#### 3.5.1 Changes in Dietary Intake

There were statistically significant reductions overall from baseline to 8 weeks for %E from fat, %E from saturated fat and %E from non‐core foods in the intervention group compared to the control group (−4.87%/day [95% CI: −8.04, −1.70], *p* = 0.005), (−1.77%/day [95% CI: −3.33, −0.20], *p* = 0.03), and (−9.20%/day [95% CI: −17.53, −0.87], *p* = 0.03) respectively. There were statistically significant reductions for total daily energy (kJ), %E from fat, %E from saturated fat, %E from monounsaturated fat, %E from non‐core foods and total trans fats and sodium (*p* < 0.05) for both groups by time.

#### 3.5.2 Changes in FA Symptoms

Changes in FA symptom scores from baseline to *8 weeks* were significantly reduced by time (*p* < 0.001), but were not statistically significant for group by time.

#### 3.5.3 Changes in Sleep Quality and Physical Activity

Differences in sleep quality and total physical activity were not statistically significant for group by time, but were significant (*p* < 0.05) by time. This indicates that from baseline to 8 weeks, sleep quality improved, and there was a significant increase in physical activity and strength training for both groups.

#### 3.5.4 Changes in Mental Health Outcomes

Changes in mental health outcomes (depression, anxiety or stress) from baseline to 8 weeks were significantly reduced by time for stress (*p* < 0.05) but not for depression or anxiety. All three mental health outcomes were not statistically significant for group by time.

#### 3.5.5 Changes in CVD Blood Outcomes

Thirty‐two (71.1%) completed their blood test at baseline (*n* = 15 intervention group, *n* = 17 control group). At the 8‐week follow‐up timepoint, 24 of the 32 participants completed their blood test (75%) (*n* = 12 intervention group, *n* = 12 control group). Changes in triglycerides were statistically significant (*p* = 0.02) by time from baseline to 8 weeks for both groups. All other outcomes were not statistically significant by time or group by time.

At baseline, 20 participants (62.5%) had CVD blood test results outside of the reference ranges (*n* = 10 intervention group, *n* = 10 control group). The number of participants with CVD blood test results outside of the reference ranges at the 8‐week timepoint was *n* = 9 (37.5%) (*n* = 5 intervention group, *n* = 4 control group).

## 4 Discussion

This study set out to determine whether an online programme aimed at improving CVD risk factors was feasible in young Australian adults with addictive eating behaviours. Results demonstrate that the majority of participants (66.7%) randomised to the intervention group completed all five telehealth sessions. Additionally, once the first telehealth session was booked, participants from the intervention group were more likely to adhere to the programme schedules, indicating considerable interest in the programme, with 100% complying with the telehealth schedule for sessions one and two, followed by 75% for session three and 85% for sessions four and five, respectively. Given this was a feasibility study, and the reported difficulties in recruiting and retaining young adults in health interventions (e.g., transient living arrangements, and a failure to recognise health problems) [70, 71, 72], the 69.8% retention rate reported is comparable to previous addictive eating studies with retention rates ranging from 74% to 79% [33, 34, 35] and provides a good foundation to base future effectiveness studies.

The post‐study interview provided valuable information regarding programme feasibility and acceptability. All participants who completed the interviews (*n* = 21) reported an overall positive experience, including duration, content, website access and ease of use. Additionally, 15 of the 16 participants indicated that text messages/email for correspondence were useful for young adults. These positive results align with a (2024) systematic review of e‐health dietary interventions (*n* = 36 studies) that reported better engagement and study retention with young adults via notifications or text [73]. It is important to note, however, that these findings are based on interviews representing 56.8% of participants. As such, the positive feedback reported may not fully reflect the views of all participants, and the potential for response bias should be considered when interpreting these findings.

The percentage of intervention group participants who accessed the website at least once compared to the control group was 95.2% versus 18.8% respectively. The reason for this disparity is unclear, as participants were not asked why they did or did not access the website. The review by Lemstra et al. [74] (*n* = 27 studies) found the main factors promoting study adherence comprised interventions that offered supervising attendance, social support, and a focus on dietary modification. Therefore, one plausible reason for the high adherence to scheduled telehealth sessions and greater website access from the intervention group participants may be due to the ongoing support and engagement through text and email reminders to access the website and a focus on eating behaviours provided during the programme, which was not offered to participants in the control group. To increase website usage, future iterations of the TRACE programme should prompt participants in the control group to access the website via reminder emails and text messages.

Website ‘dwell’ time (i.e., the time spent on each webpage) for four of the nine webpages was found to meet industry benchmarks of 2–4 min or more [75]. Although dwell time measured in this study indicated a moderate to high interaction and acceptability of the study website and usage, suggesting that participants found the content valuable and relevant, researchers were unable to confirm whether users truly engaged with the content. Using website usage data (such as the number of logins and dwell time) as a proxy for engagement may neglect the fact that website use may not necessarily mean effective use [76]. To truly understand the degree and extent of website engagement and usage data, future e‐health studies should combine both an in‐depth subjective measure (e.g., qualitative interviews with participants around website engagement) and objective measures such as dwell time, number of visitors and exit rate [77].

In addition to retention rate, telehealth session adherence, and overall programme acceptability, there was a moderate number of participants completing their blood tests. Collecting objective markers (i.e., venous blood sample) as part of clinical trials does place a large burden on the participant. Recent research suggests that capillary finger‐prick tests may offer an alternative, therefore lowering this burden [78, 79]. Although most CVD blood biomarkers were not significantly different between groups or by time, potentially due to the study being underpowered and the short turnaround of follow‐up blood tests (i.e., 8 weeks apart), there were a number of participants found to have results outside of the reference ranges. Of those participants who had blood tests, a considerable number (62.5%) had readings outside of the reference ranges at baseline, as indicated by elevated fasting glucose or blood fats. By the 8‐week timepoint, this number reduced to 37.5%. Based on the blood test results for this study, there appears to be a need to further explore CVD blood biomarkers and increase knowledge around CVD risk factors in a larger population of young adults experiencing addictive eating behaviours.

Similar lifestyle pilot studies designed to improve health outcomes in young adults have also reported positive feasibility outcomes. Both the HEYMAN [80] intervention – a healthy lifestyle programme for young men, and the DHC [81] trial – a lifestyle intervention utilising social media to target health behaviours in women aged 18–24, reported positive participant acceptability and satisfaction in their respective programmes. However, unlike the HEYMAN intervention, which reported significant improvements in cholesterol, changes in cardiometabolic biomarkers were limited in this current study, possibly due to the small sample size and the short time frame between baseline and follow‐up blood tests. Similar to the HEYMAN intervention which reported improvements in dietary outcomes such as core and non‐core foods [80], results of the current study indicated a significant improvement in a number of dietary outcomes related to CVD risk (namely %E from fat, saturated fat and non‐core foods) in the intervention group compared to the control group at 8 weeks. This also warrants further investigation in a larger, more adequately powered trial. Additionally, the improvements in total sodium and trans fats (both of which are dietary risk factors for CVD) as well as FA symptoms, sleep quality, physical activity and mental health outcomes (specifically stress) reported in both groups over time suggests that the ‘TRACE‐young adults’ programme has the potential to positively change young adults' health behaviours through increasing awareness around addictive eating behaviours. This is in contrast to the majority of CVD interventions, which focus on improving diet and reducing CVD risk through nutrient‐specific advice and education [10, 82, 83]. It was interesting to note that improvements in FA symptoms, sleep quality and physical activity were significant in both groups. It may be the fact that eligible participants felt ‘validated’, which confirmed their addictive eating behaviours were ‘real’ and therefore began to incorporate healthier habits irrespective of group allocation.

The differential impact of the intervention across outcomes may be partly attributed to its core focus. While the intervention targeted multiple CVD risk factors, the content and delivery placed more emphasis on improving dietary intake and addictive eating behaviours. As such, changes in food‐related outcomes may have been more pronounced due to the depth and frequency of related content. Additionally, the personalised nature of the intervention meant that some participants may have focused more intensively on dietary change, while others may have engaged more with content related to physical activity or sleep, depending on their individual priorities and readiness to change. Furthermore, a large proportion of the sample (84.9%) was classified as sufficiently active at baseline, leaving limited scope for measurable improvement in physical activity. In terms of improvements in mental health, the 8‐week duration of the programme may not have been sufficient to produce clinically meaningful changes, particularly in outcomes such as depression or anxiety, which often require longer‐term support and intervention [84].

### 4.1 Limitations

Although the results of this study are promising, there are limitations to consider. The current study was a feasibility study, with participants being volunteers and likely motivated to change, while those reporting eating disorders or severe mental ill‐health (a population difficult to motivate to make lifestyle changes) were excluded from the study. The over‐representation of females (79.2%) is unlikely to be representative of the general population, and the use of self‐reported measures may have contributed to recall bias. While adherence was measured based on session attendance, the authors acknowledge that attendance alone may not fully capture participants' engagement with or enactment of the intervention content. Future studies should consider incorporating more robust adherence metrics, such as fidelity checklists, to comprehensively evaluate intervention uptake. The small sample size for blood biomarker analysis was likely underpowered to detect significant changes. While some improvements were observed over time, larger studies with extended follow‐up are needed to confirm effects on cardiometabolic outcomes in young adults. Lastly, while moderate to high dwell time on the majority of website pages was interpreted as a potential indicator of interest and usability, this metric alone may not fully reflect meaningful engagement with the digital content. Without more detailed analytics (e.g., dwell time, number of visitors and exit rate), it is difficult to determine the depth or quality of interaction. Additionally, engagement with the text message component could not be meaningfully assessed, as the messages were one‐way—delivered by the research team as reminders without a mechanism for participant response or interaction. As such, conclusions about participant engagement with the website and SMS content should be interpreted with caution.

## 5 Conclusion

A CVD intervention for young adults with addictive eating behaviours was found to be feasible and demonstrated some improvements in several dietary outcomes. Retention was comparable to other interventions, the programme had good adherence to telehealth schedules, blood test compliance and positive feedback showed a willingness to participate in an intervention for reducing CVD risk factors with a focus on eating behaviours. This provides support for the conduct of a larger, fully‐powered RCT, but with some changes to research procedures, including more comprehensive fidelity checklists.

## Author Contributions

**Mark A. Leary:** conceptualisation, data curation and analysis, project administration, software, writing – original draft. **Janelle A. Skinner:** conceptualisation, project administration, writing – review and editing. **Melinda J. Hutchesson:** conceptualisation, writing – review and editing. **Megan Teychenne:** conceptualisation, writing – review and editing. **Megan C. Whatnall:** conceptualisation, writing – review and editing. **Phillipa J. Hay:** conceptualisation, writing – review and editing. **Tracy L. Burrows:** conceptualisation, funding acquisition, investigation, methodology, supervision, writing – review and editing.

## Ethics Statement

The study was approved by The University of Newcastle Human Research Ethics Committee (H‐2022‐0386) and was registered as a clinical trial with the Australian New Zealand Clinical Trials Registry before commencing recruitment (ACTRN12623001079639).

## Consent

The authors have nothing to report.

## Conflicts of Interest

The authors declare no conflicts of interest.

## Peer Review

The peer review history for this article is available at https://www.webofscience.com/api/gateway/wos/peer-review/10.1111/jhn.70102.

## Supporting information


**Additional Table S1:** Overview of intervention sessions of the TRACE programme.

**Additional Table S2:** Qualitative content analysis from participants completing the feasibility and acceptability interview.

**Additional Table S3:** Changes in dietary variables within and between groups (baseline to 8‐weeks with (95% confidence intervals)) with under‐reporters at baseline excluded.

## Data Availability

The data sets used and/or analysed during the current study are available from the corresponding author on reasonable request.
