# Supplementary material for: Feasibility and Preliminary Efficacy of an Online Cardiovascular Disease Prevention Randomised Controlled Trial Targeting Addictive and Compulsive Overeating Among Australian Young Adults
Source: J Hum Nutr Diet. 2025 Jul 28;38(4):e70102. doi: 10.1111/jhn.70102 (PMC12304629; doi:10.1111/jhn.70102)
Supplement: Supplementary file 1 — Additional Table S1: Overview of intervention sessions of the TRACE programme. [file JHN-38-0-s002.docx]

Additional Table S1: Overview of intervention sessions of the TRACE programme.

| **Session** | **Session aims** |
| --- | --- |
| **Session 1**  **(Week 1 – 45mins)**  **Personality** | - Introduce the intervention - Determine participant’s main concerns with their food intake - Provide feedback baseline scores of addictive eating (*Yale Food Addiction Scale 2.0*) - Discuss what this means for the participant when attempting and preparing to make changes to improve CVD risk factors - Provide feedback on major personality trait (*Substance Use Risk Profile Scale*) [Anxiety proneness, Depression proneness, Impulsivity or Sensation seeking] - Discuss how personality traits may relate to food intake and addictive eating, and what this means for the participant - Discuss coping strategies based on personality traits and complete ‘Urge Surfing’ activity - Introduce ‘Distraction List’ - Set homework task: choose and practice 2 coping strategy exercises - Provide session summary |
| **Session 2**  **(Week 2 – 45mins)**  **Food/Physical Activity** | - Review session 1 - Check in for episodes of overeating and discuss progress with coping strategies - Provide feedback of dietary intake (*Australian Eating Survey – Heart version*) - *Optional: discuss alcohol intake* - Provide feedback of physical activity (*Active Australia Survey*) - Develop 4 goals for heart health using *SMARTER Goal Checklist*  1. Positive – increase core foods 2. Reduction – decrease non-core foods 3. ‘Eating awareness’ – using strategies to delay or halt overeating 4. Physical activity – using strategies for being more active  - Discuss enabler/barriers to making changes to eating habits and physical activity levels - Set homework task: complete *Triggers for overeating* worksheet - Provide session summary |
| **Session 3**  **(Week 4 – 30mins)**  **Skills** | - Review session 2 - Assess progress with SMARTER goals (nutrition and physical activity) - Check in for episodes of overeating - Discuss 'Triggers for Overeating' - Create strategies to overcome triggers, building on previous personality based coping strategies and ‘Practical Strategies to Achieve Goals’ - Discuss and determine a ‘food line’ to identify when eating is no longer enjoyable or not tasting food - Set homework task: complete *Mood Monitor* worksheet - Provide session summary |
| **Session 4**  **(Week 6 – 30mins)**  **Confidence** | - Review session 3 - Check in for episodes of overeating - Discuss progress with Foodline plan/goals and enablers/barriers - Discuss ‘Mood Monitor’ and explore emotions that participant has difficulty coping with - Explore coping strategies for difficult emotions - Discuss importance of sleep, caffeine intake, and responsible intake of alcohol for emotional health and set goals where appropriate - Provide feedback for sleep (*Pittsburgh Sleep Quality Index*) and set goals to improve sleep where appropriate - Assess progress with food/physical activity goals - Set homework task: practice implementation of ‘Coping skill plan’ to achieve goals - Provide session summary |
| **Session 5**  **(Week 8 – 15mins)**  **Moving forward** | - Review session 4 - Check in/briefly problem solve and encourage to continue with goals and strategies - Discuss topics from previous sessions (participant led) - Reassess confidence to achieve goals - Provide final *Addictive Eating Action Plan* |
