# Supplementary material for: Feasibility and Preliminary Efficacy of an Online Cardiovascular Disease Prevention Randomised Controlled Trial Targeting Addictive and Compulsive Overeating Among Australian Young Adults
Source: J Hum Nutr Diet. 2025 Jul 28;38(4):e70102. doi: 10.1111/jhn.70102 (PMC12304629; doi:10.1111/jhn.70102)
Supplement: Supplementary file 2 — Additional Table S2: Qualitative content analysis from participants completing the feasibility and acceptability interview. [file JHN-38-0-s001.docx]

Additional Table S2: Qualitative content analysis from participants completing the feasibility and acceptability interview.

| **Feasibility and acceptability questions**  **(intervention and waitlist control group n = 21)** | **Participant Response** | **Number of participants** |
| --- | --- | --- |
| Appropriateness of recruitment materials (e.g., suitability, right language or pitch) | Social media posts and recruitment flyers were clear, suitable and effective | 18 |
| Appropriateness of study surveys (e.g., easy to understand, easy to complete, time taken) | Easy to understand and complete - Yes | 21 |
| Overall experience with the study | Overall positive | 21 |
|  | Gained awareness of habits and helpful strategies | 7 |
|  | Felt supported and comfortable | 5 |
|  | Blood tests were difficult to organise | 2 |
| **Feasibility and acceptability questions**  **(intervention group only n = 16)** |  |  |
| **Telehealth sessions** |  |  |
| Appropriateness of telehealth sessions (e.g., telehealth sessions easier than attending in person, duration appropriate, availability of session times suitable?) | Yes | 16 |
| Did you find the number of telehealth sessions offered sufficient? | Yes | 9 |
|  | Would have preferred more sessions | 6 |
| **Program content** |  |  |
| Did you find the information provided in the sessions useful, appropriate and easy to understand? | Yes | 16 |
| In what way did the information help you change your behaviours? | Able to use coping strategies in daily life | 11 |
|  | Increased awareness of eating behaviours | 7 |
|  | Goal setting and reflecting very useful | 7 |
| Did you find the addictive eating action plan you received after the sessions useful? | Yes | 14 |
| Did you find the study workbook useful? | Overall, yes | 15 |
| Was the workbook easy to use? | Overall, yes | 13 |
| Did you like the presentation and layout of the workbook? | Overall, yes | 15 |
| **Dietitian** |  |  |
| Did you find the dietitian was knowledgeable? | Yes | 16 |
| Did you find the dietitian had good communication skills? | Yes | 16 |
| Did you feel comfortable asking the dietitian questions? | Yes | 16 |
| **Website** |  |  |
| Did you find the website useful? | Overall, yes | 12 |
| Was the website easy to use? | Yes | 16 |
| Did you like the presentation and layout of the website? | Yes | 16 |
| Overall, did you find the website complimented the information provided to you in the workbook, and also by the dietitian in the telehealth sessions? | Yes | 12 |
| **Correspondence during the program** |  |  |
| Did you find receiving SMS reminders throughout the study useful? | Yes | 15 |
| How do you feel about the number of text messages you received | Good amount | 13 |
| Did you find the emails easy to understand? | Yes | 8 |
| **Satisfaction with the intervention** |  |  |
| Overall, how would you rate your satisfaction with the intervention? | Very good (9 or 10 out of 10) | 13 |
|  | Good (6 or 7 out of 10) | 3 |
