# Supplementary material for: Feasibility and Preliminary Efficacy of an Online Cardiovascular Disease Prevention Randomised Controlled Trial Targeting Addictive and Compulsive Overeating Among Australian Young Adults
Source: J Hum Nutr Diet. 2025 Jul 28;38(4):e70102. doi: 10.1111/jhn.70102 (PMC12304629; doi:10.1111/jhn.70102)
Supplement: Supplementary file 3 — Additional Table S3: Changes in dietary variables within and between groups (baseline to 8‐weeks with (95% confidence intervals)) with under‐reporters at baseline excluded. [file JHN-38-0-s003.docx]

Additional Table S3: Changes in dietary variables within and between groups (baseline to 8-weeks with (95% confidence intervals)) with under-reporters at baseline excluded.

|  | **Difference over time** | |  | **Difference between groups over time** | |
| --- | --- | --- | --- | --- | --- |
| **Variable** | **Intervention group** | **Control group** | **Time p** | **Intervention-Control** | **Group x Time p** |
| **Nutrient** |  |  |  |  |  |
| Total Energy (kJ) | -2073 (-3987, -160) | -1308 (-3238, 623) | **<0.05** | -765 (-3483, 1953) | 0.58 |
| %E from fat | -3.38 (-6.33, -0.42) | 1.99 (-0.98, 4.95) | **<0.05** | -5.36 (-9.55, -1.18) | **<0.05** |
| %E from saturated fat | -2.02 (-3.53, -0.52) | 0.54 (-0.98, 2.07) | **<0.05** | -2.57 (-4.71, -0.43) | **<0.05** |
| %E from monounsaturated fat | -1.63 (-2.96, -0.30) | 0.38 (-0.95, 1.72) | **<0.05** | -2.02 (-3.90, -0.14) | 0.05 |
| %E from polyunsaturated fat | 0.06 (-1.09, 1.21) | 0.94 (-0.21, 2.09) | 0.92 | -0.88 (-2.50, 0.74) | 0.30 |
| Total Trans fat (mg) | -486 (-937, -35) | -84 (-537, 368) | **<0.05** | -402 (-1041, 237) | 0.23 |
| Total Sodium (mg) | -614 (-1066, -161) | -221 (-678, 235) | **<0.05** | -392 (-1034, 250) | 0.24 |
| Total Fibre (g) | 2.26 (-4.90, 9.43) | -5.60 (-12.84, 1.65) | 0.54 | 7.86 (-2.33, 18.05) | 0.33 |
| %E from non-core foods | -13.13 (-19.56, -6.71) | -0.28 (-6.78, 6.23) | **<0.001** | -12.85 (-21.99, -3.71) | **<0.05** |

Note: Boldface indicates statistical significance
